# Bis{μ-1,3-bis­[2-(5-bromo-2-oxidobenzyl­idene­amino)ethyl]-2-(5-bromo-2-oxido­phenyl)-1,3-imidazolidine}dineo­dymium(III) *N*,*N*-dimethyl­formamide hexa­solvate

**DOI:** 10.1107/S1600536809047564

**Published:** 2009-11-21

**Authors:** Qing-Fan Xie, Miao-Ling Huang, Yan-Min Chen

**Affiliations:** a Department of Chemistry and Science of Life, Quanzhou Normal University, Fujian 362000, People’s Republic of China

## Abstract

In the title centrosymmetric dinuclear complex, [Nd_2_(C_27_H_24_Br_3_N_4_O_3_)_2_]·6C_3_H_7_NO, the Nd^III^ ion is coordinated in a slightly distorted square-anti­prismatic geometry by four N atoms and four O atoms from two centrosymmetrically-related 1,3-bis­[2-(5-bromo-2-oxidobenzyl­amino)eth­yl]-2-(5-bromo-2-oxidophen­yl)-1,3-imidazolidine ligands. The Nd⋯Nd separation is 4.5012 (12) Å.

## Related literature

For general background to tripodal ligands, see: Bian *et al.* (2008 ▶); Palaniandavar *et al.* (2006 ▶); Velusamy *et al.* (2004 ▶). For related structures, see: Fondo *et al.* (2005 ▶); Xie *et al.* (2009 ▶); Yang *et al.* (1995 ▶).
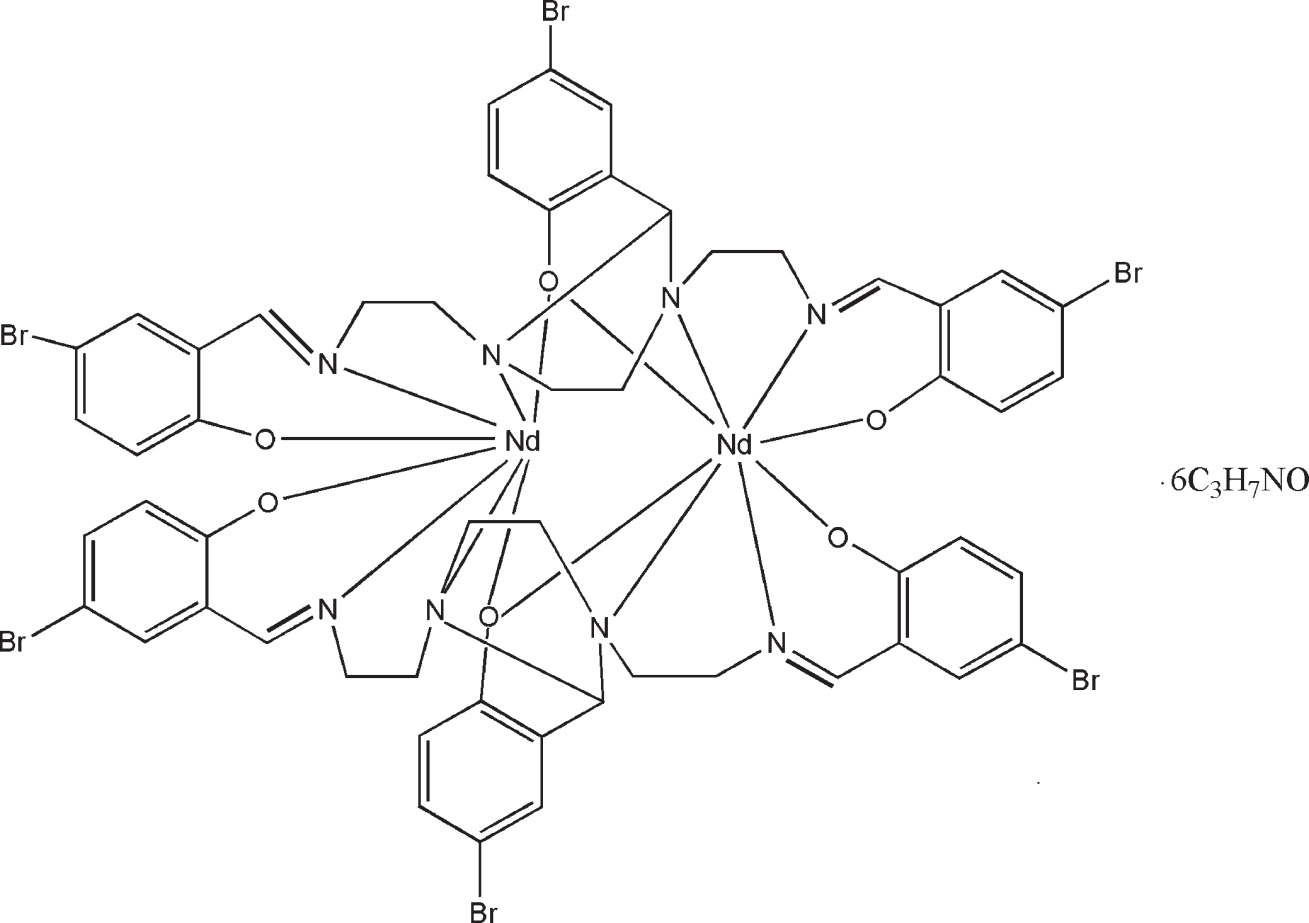


## Experimental

### 

#### Crystal data

[Nd_2_(C_27_H_24_Br_3_N_4_O_3_)_2_]·6C_3_H_7_NO
*M* *_r_* = 2111.46
Monoclinic, 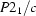

*a* = 14.624 (6) Å
*b* = 22.460 (4) Å
*c* = 13.663 (4) Å
β = 101.133 (6)°
*V* = 4403 (2) Å^3^
*Z* = 2
Mo *K*α radiation
μ = 3.95 mm^−1^
*T* = 296 K
0.25 × 0.23 × 0.22 mm

#### Data collection

Bruke APEXII CCD diffractometer
Absorption correction: multi-scan (*SADABS*; Sheldrick, 1996 ▶) *T*_min_ = 0.438, *T*_max_ = 0.477
25933 measured reflections
8078 independent reflections
5119 reflections with *I* > 2σ(*I*)
*R*_int_ = 0.057

#### Refinement

*R*[*F* ^2^ > 2σ(*F* ^2^)] = 0.046
*wR*(*F* ^2^) = 0.120
*S* = 1.02
8078 reflections
484 parameters
62 restraints
H-atom parameters constrained
Δρ_max_ = 0.84 e Å^−3^
Δρ_min_ = −0.78 e Å^−3^

### 

Data collection: *APEX2* (Bruker, 2007 ▶); cell refinement: *SAINT* (Bruker, 2007 ▶); data reduction: *SAINT*; program(s) used to solve structure: *SHELXS97* (Sheldrick, 2008 ▶); program(s) used to refine structure: *SHELXL97* (Sheldrick, 2008 ▶); molecular graphics: *SHELXTL* (Sheldrick, 2008 ▶); software used to prepare material for publication: *SHELXTL*.

## Supplementary Material

Crystal structure: contains datablocks global, I. DOI: 10.1107/S1600536809047564/hy2250sup1.cif

Structure factors: contains datablocks I. DOI: 10.1107/S1600536809047564/hy2250Isup2.hkl

Additional supplementary materials:  crystallographic information; 3D view; checkCIF report

## Figures and Tables

**Table 1:** Selected bond lengths (Å)

Nd1—O1^i^	2.341 (4)
Nd1—O2	2.344 (4)
Nd1—O3	2.448 (4)
Nd1—O3^i^	2.467 (4)
Nd1—N1^i^	2.616 (5)
Nd1—N2^i^	2.864 (4)
Nd1—N3	2.896 (5)
Nd1—N4	2.627 (5)

Symmetry code: (i) 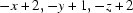.

**Fig. 1.**
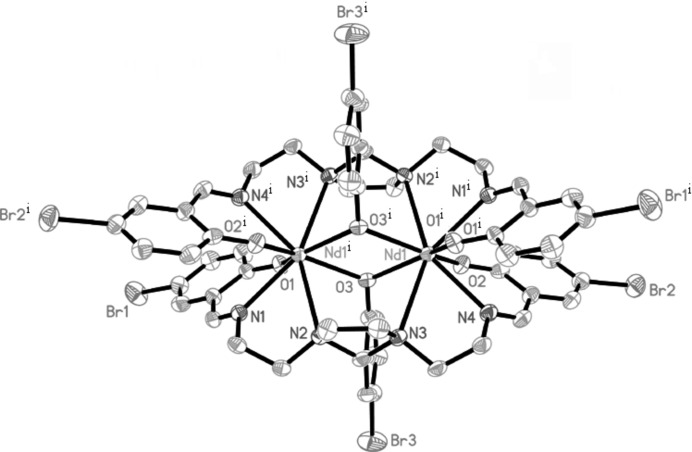
The molecular structure of the title compound. Displacement ellipsoids are drawn at the 30% probability level. [Symmetry codes: (i) -x+2, -y+1, -z+2.]

## supplementary crystallographic information

### Comment

As the rare-earth ions have unique electronic structures and bonding characteristics, the formation of complexes has managed to maintain its unique electromagnetic nature of light. Tripodal ligand has a semi-rigid structure. It can provide a number of service sites to form thermodynamically stable complexes, and its three side chains are free to flip to form a suitable cavity size to include different guest molecules or ions. Furthermore, the researches of tripodal ligands and their complexes are very active at present (Bian *et al.*, 2008; Palaniandavar *et al.*, 2006; Velusamy *et al.*, 2004).

The molecular diagram of the title compound is presented in Fig. 1. The structure is composed of a dimeric [Nd_2_(brapi)_2_] molecule [brapi = 2-(2'-hydroxy-5'-bromophenyl)-1,3-bis[3'-aza-4'-(2''-hydroxy- 5''-bromophenyl)-prop-4'-en-1'-yl]-1,3-imidazolidine], with eight-coordinated Nd^III^ ions linked by two bridging O atoms from the phenolic hydroxyl groups, and six N,N-dimethylformamide (DMF) molecules. The coordination geometry around the Nd^III^ ion may be described as distorted square antiprismatic, with one square plane being defined by O2, O3, N3, N4 [the torsion angle is 3.07 (17)°] and the other defined by O1, O3, N2, N1 [the torsion angle is 2.03 (16)°]. The coordination to the metal of the O atoms and N atoms results in the bond lengths of C—O [1.328 (8)–1.364 (7) Å] and C—N [1.301 (7)–1.527 (7) Å] are longer than those in the ligand (Fondo *et al.*, 2005) and in complexes [Ce_2_(brapi)_2_].2DMF (Xie *et al.*, 2009) and [La_2_(brapi)_2_].2CHCl_3_ (Yang *et al.*, 1995). The bond lengths of Nd—O (Table 1) are similar to those in the complexes [Ce_2_(brapi)_2_].2DMF and [La_2_(brapi)_2_].2CHCl_3_. It can be seen that there is an intermolecular C—H···O hydrogen bond between the DMF molecule and the ligand. The solvent DMF molecules play a role in stablizing the crystal structure.

### Experimental

A mixture of H_3_brapi (1 mmol), Nd(NO_3_)_3_.6H_2_O (1 mmol) and DMF (12 ml) was sealed in a 18 ml Teflon-lined stainless steel reactor and heated in an oven at 353 K for 5 d, and then slowly cooled to room temperature. Orange hexagonal prism crystals of the title complex were collected.

### Refinement

H atoms were placed at calculated positions (C—H = 0.93–0.98 Å) and were allowed to ride on their parent atoms, with *U*_iso_(H) = 1.2(1.5 for methyl)*U*_eq_(C).

### Crystal data

**Table:**

[Nd_2_(C_27_H_24_Br_3_N_4_O_3_)_2_]·6C_3_H_7_NO	*F*(000) = 2092
*M**_r_* = 2111.46	*D*_x_ = 1.593 Mg m^−^^3^
Monoclinic, *P*2_1_/*c*	Mo *K*α radiation, λ = 0.71073 Å
Hall symbol: -P 2ybc	Cell parameters from 3640 reflections
*a* = 14.624 (6) Å	θ = 2.3–20.3°
*b* = 22.460 (4) Å	µ = 3.95 mm^−^^1^
*c* = 13.663 (4) Å	*T* = 296 K
β = 101.133 (6)°	Prism, orange
*V* = 4403 (2) Å^3^	0.25 × 0.23 × 0.22 mm
*Z* = 2	

### Data collection

**Table:**

Bruke APEXII CCD diffractometer	8078 independent reflections
Radiation source: fine-focus sealed tube	5119 reflections with *I* > 2σ(*I*)
graphite	*R*_int_ = 0.057
φ and ω scans	θ_max_ = 25.5°, θ_min_ = 2.3°
Absorption correction: multi-scan (*SADABS*; Sheldrick, 1996)	*h* = −17→13
*T*_min_ = 0.438, *T*_max_ = 0.477	*k* = −27→27
25933 measured reflections	*l* = −16→16

### Refinement

**Table:**

Refinement on *F*^2^	Primary atom site location: structure-invariant direct methods
Least-squares matrix: full	Secondary atom site location: difference Fourier map
*R*[*F*^2^ > 2σ(*F*^2^)] = 0.046	Hydrogen site location: inferred from neighbouring sites
*wR*(*F*^2^) = 0.120	H-atom parameters constrained
*S* = 1.02	*w* = 1/[σ^2^(*F*_o_^2^) + (0.0539*P*)^2^ + 1.2941*P*] where *P* = (*F*_o_^2^ + 2*F*_c_^2^)/3
8078 reflections	(Δ/σ)_max_ = 0.001
484 parameters	Δρ_max_ = 0.84 e Å^−^^3^
62 restraints	Δρ_min_ = −0.78 e Å^−^^3^

### Fractional atomic coordinates and isotropic or equivalent isotropic displacement parameters (Å^2^)

**Table:**

	*x*	*y*	*z*	*U*_iso_*/*U*_eq_	
Nd1	1.09682 (2)	0.516119 (15)	0.91542 (2)	0.04309 (12)	
O1	0.8295 (3)	0.57032 (18)	1.1258 (3)	0.0549 (12)	
O2	1.1124 (3)	0.61662 (18)	0.8752 (3)	0.0551 (11)	
O3	0.9518 (3)	0.55074 (16)	0.9608 (3)	0.0453 (10)	
O4	0.7262 (6)	0.4034 (4)	0.5864 (6)	0.143 (3)	
O5	0.9895 (8)	0.6783 (5)	0.4013 (8)	0.214 (4)	
O6	0.4457 (4)	0.4441 (2)	0.1416 (4)	0.0840 (16)	
N1	0.7226 (4)	0.4679 (2)	1.0519 (4)	0.0458 (13)	
N2	0.8233 (3)	0.44465 (19)	0.8871 (3)	0.0408 (12)	
N3	0.9378 (4)	0.4633 (2)	0.7851 (3)	0.0451 (13)	
N4	1.0965 (4)	0.5268 (2)	0.7239 (4)	0.0512 (14)	
N5	0.6314 (6)	0.4532 (5)	0.4580 (6)	0.111 (3)	
N6	0.9545 (6)	0.6979 (3)	0.5551 (7)	0.109 (2)	
N7	0.4382 (5)	0.3491 (3)	0.0826 (4)	0.0726 (18)	
Br1	0.50063 (7)	0.64057 (4)	1.32131 (8)	0.1009 (3)	
Br2	1.33362 (7)	0.75756 (4)	0.61348 (6)	0.0850 (3)	
Br3	0.65268 (7)	0.68123 (4)	0.66156 (8)	0.1146 (4)	
C1	0.7576 (5)	0.5835 (3)	1.1687 (5)	0.0470 (16)	
C2	0.6724 (5)	0.5486 (3)	1.1534 (5)	0.0490 (16)	
C3	0.5970 (5)	0.5677 (3)	1.1966 (5)	0.0617 (19)	
H3	0.5415	0.5463	1.1833	0.074*	
C4	0.6030 (5)	0.6180 (3)	1.2590 (5)	0.0628 (19)	
C5	0.6870 (6)	0.6513 (3)	1.2762 (5)	0.067 (2)	
H5	0.6925	0.6845	1.3176	0.080*	
C6	0.7615 (5)	0.6349 (3)	1.2319 (5)	0.068 (2)	
H6	0.8154	0.6579	1.2437	0.081*	
C7	0.6587 (5)	0.4961 (3)	1.0878 (5)	0.0477 (16)	
H7	0.5982	0.4816	1.0702	0.057*	
C8	0.6907 (5)	0.4201 (3)	0.9770 (4)	0.0504 (17)	
H8A	0.6237	0.4152	0.9677	0.060*	
H8B	0.7201	0.3826	0.9996	0.060*	
C9	0.7180 (4)	0.4389 (3)	0.8787 (4)	0.0477 (16)	
H9A	0.6943	0.4097	0.8277	0.057*	
H9B	0.6889	0.4768	0.8578	0.057*	
C10	0.8680 (5)	0.3849 (3)	0.8719 (5)	0.0569 (18)	
H10A	0.8961	0.3677	0.9357	0.068*	
H10B	0.8215	0.3574	0.8374	0.068*	
C11	0.9415 (5)	0.3967 (3)	0.8102 (5)	0.0585 (19)	
H11A	0.9291	0.3732	0.7494	0.070*	
H11B	1.0027	0.3861	0.8475	0.070*	
C12	0.9465 (5)	0.4751 (3)	0.6783 (4)	0.0545 (18)	
H12A	0.9173	0.5129	0.6572	0.065*	
H12B	0.9132	0.4443	0.6359	0.065*	
C13	1.0481 (5)	0.4765 (3)	0.6639 (5)	0.0579 (18)	
H13A	1.0788	0.4392	0.6858	0.070*	
H13B	1.0501	0.4821	0.5939	0.070*	
C14	1.1301 (5)	0.5694 (3)	0.6763 (5)	0.0592 (19)	
H14	1.1281	0.5637	0.6085	0.071*	
C15	1.1575 (5)	0.6467 (3)	0.8157 (5)	0.0525 (17)	
C16	1.1705 (5)	0.6250 (3)	0.7199 (5)	0.0533 (17)	
C17	1.2211 (5)	0.6604 (3)	0.6599 (5)	0.0606 (19)	
H17	1.2278	0.6466	0.5975	0.073*	
C18	1.2598 (5)	0.7148 (3)	0.6938 (5)	0.0606 (19)	
C19	1.2454 (5)	0.7379 (3)	0.7859 (5)	0.0606 (19)	
H19	1.2693	0.7750	0.8076	0.073*	
C20	1.1950 (5)	0.7048 (3)	0.8445 (5)	0.0584 (18)	
H20	1.1855	0.7210	0.9044	0.070*	
C21	0.8421 (5)	0.4808 (3)	0.7978 (4)	0.0476 (16)	
H21	0.7977	0.4681	0.7384	0.057*	
C22	0.8863 (4)	0.5795 (3)	0.8925 (4)	0.0472 (16)	
C23	0.8291 (4)	0.5476 (3)	0.8122 (4)	0.0449 (15)	
C24	0.7604 (5)	0.5790 (3)	0.7447 (5)	0.0568 (18)	
H24	0.7224	0.5586	0.6931	0.068*	
C25	0.7488 (5)	0.6406 (3)	0.7547 (6)	0.068 (2)	
C26	0.8068 (5)	0.6723 (3)	0.8294 (6)	0.071 (2)	
H26	0.8011	0.7134	0.8334	0.086*	
C27	0.8741 (5)	0.6418 (3)	0.8990 (5)	0.0614 (19)	
H27	0.9113	0.6630	0.9502	0.074*	
C28	0.6559 (9)	0.4070 (6)	0.5211 (8)	0.117 (4)	
H28	0.6154	0.3747	0.5142	0.140*	
C29	0.5455 (9)	0.4509 (6)	0.3801 (10)	0.199 (7)	
H29A	0.5119	0.4150	0.3876	0.298*	
H29B	0.5619	0.4513	0.3153	0.298*	
H29C	0.5071	0.4847	0.3867	0.298*	
C30	0.6919 (8)	0.5047 (5)	0.4636 (8)	0.129 (4)	
H30A	0.7489	0.4969	0.5100	0.193*	
H30B	0.6614	0.5386	0.4856	0.193*	
H30C	0.7056	0.5126	0.3989	0.193*	
C31	1.0027 (9)	0.7059 (5)	0.4841 (9)	0.128 (3)	
H31	1.0503	0.7340	0.4955	0.153*	
C32	0.9706 (8)	0.7371 (5)	0.6416 (7)	0.127 (3)	
H32A	0.9884	0.7138	0.7012	0.191*	
H32B	0.9145	0.7587	0.6446	0.191*	
H32C	1.0194	0.7647	0.6362	0.191*	
C33	0.8992 (10)	0.6434 (6)	0.5505 (11)	0.198 (5)	
H33A	0.8369	0.6512	0.5155	0.297*	
H33B	0.8976	0.6302	0.6170	0.297*	
H33C	0.9269	0.6131	0.5161	0.297*	
C34	0.4033 (6)	0.4034 (3)	0.0944 (5)	0.068 (2)	
H34	0.3417	0.4105	0.0639	0.081*	
C35	0.3825 (8)	0.3037 (4)	0.0193 (7)	0.127 (4)	
H35A	0.3209	0.3188	−0.0050	0.190*	
H35B	0.3788	0.2683	0.0578	0.190*	
H35C	0.4116	0.2945	−0.0361	0.190*	
C36	0.5326 (6)	0.3332 (4)	0.1312 (7)	0.103 (3)	
H36A	0.5697	0.3262	0.0816	0.154*	
H36B	0.5310	0.2977	0.1701	0.154*	
H36C	0.5594	0.3651	0.1741	0.154*	

### Atomic displacement parameters (Å^2^)

**Table:**

	*U*^11^	*U*^22^	*U*^33^	*U*^12^	*U*^13^	*U*^23^
Nd1	0.0450 (2)	0.0465 (2)	0.03667 (18)	−0.00268 (17)	0.00517 (14)	−0.00282 (15)
O1	0.049 (3)	0.050 (3)	0.068 (3)	−0.004 (2)	0.018 (2)	−0.007 (2)
O2	0.068 (3)	0.050 (3)	0.049 (3)	0.001 (2)	0.017 (2)	0.003 (2)
O3	0.047 (3)	0.045 (2)	0.040 (2)	0.003 (2)	0.001 (2)	−0.0026 (18)
O4	0.129 (7)	0.168 (7)	0.116 (6)	−0.006 (5)	−0.015 (5)	−0.020 (5)
O5	0.231 (8)	0.236 (8)	0.191 (6)	−0.015 (6)	0.082 (6)	−0.048 (6)
O6	0.078 (4)	0.063 (3)	0.104 (4)	−0.008 (3)	0.002 (3)	−0.014 (3)
N1	0.047 (3)	0.048 (3)	0.042 (3)	−0.002 (3)	0.008 (2)	−0.005 (2)
N2	0.040 (3)	0.041 (3)	0.041 (3)	0.002 (2)	0.006 (2)	−0.008 (2)
N3	0.052 (3)	0.045 (3)	0.038 (3)	0.003 (2)	0.010 (2)	−0.006 (2)
N4	0.057 (4)	0.055 (3)	0.042 (3)	−0.006 (3)	0.008 (3)	−0.007 (2)
N5	0.101 (7)	0.152 (8)	0.072 (5)	0.000 (6)	−0.003 (5)	−0.010 (5)
N6	0.124 (5)	0.093 (4)	0.115 (5)	−0.005 (4)	0.038 (4)	−0.018 (4)
N7	0.083 (5)	0.061 (4)	0.068 (4)	0.007 (3)	0.002 (4)	0.006 (3)
Br1	0.0759 (6)	0.1142 (8)	0.1206 (8)	0.0082 (5)	0.0388 (6)	−0.0435 (6)
Br2	0.0998 (7)	0.0696 (5)	0.0893 (6)	−0.0105 (5)	0.0274 (5)	0.0242 (4)
Br3	0.1152 (8)	0.0817 (6)	0.1245 (8)	0.0199 (6)	−0.0330 (7)	0.0376 (6)
C1	0.042 (4)	0.048 (4)	0.051 (4)	0.002 (3)	0.010 (3)	−0.001 (3)
C2	0.047 (4)	0.049 (4)	0.052 (4)	0.003 (3)	0.013 (3)	−0.006 (3)
C3	0.066 (5)	0.063 (4)	0.053 (4)	−0.003 (4)	0.002 (4)	−0.004 (3)
C4	0.061 (5)	0.065 (5)	0.065 (5)	0.008 (4)	0.018 (4)	−0.010 (4)
C5	0.078 (6)	0.054 (4)	0.070 (5)	−0.001 (4)	0.018 (4)	−0.020 (4)
C6	0.065 (5)	0.061 (5)	0.077 (5)	−0.003 (4)	0.014 (4)	−0.017 (4)
C7	0.048 (4)	0.043 (3)	0.052 (4)	−0.009 (3)	0.008 (3)	0.001 (3)
C8	0.047 (4)	0.046 (4)	0.057 (4)	−0.004 (3)	0.004 (3)	−0.012 (3)
C9	0.049 (4)	0.046 (4)	0.045 (4)	−0.004 (3)	0.003 (3)	−0.009 (3)
C10	0.071 (5)	0.042 (4)	0.061 (4)	0.002 (3)	0.019 (4)	−0.004 (3)
C11	0.064 (5)	0.044 (4)	0.064 (4)	0.001 (3)	0.005 (4)	−0.009 (3)
C12	0.064 (5)	0.063 (4)	0.033 (3)	−0.009 (3)	0.002 (3)	−0.007 (3)
C13	0.068 (5)	0.066 (4)	0.040 (3)	−0.010 (4)	0.009 (3)	−0.012 (3)
C14	0.073 (5)	0.068 (5)	0.037 (4)	0.003 (4)	0.012 (4)	−0.001 (3)
C15	0.057 (5)	0.050 (4)	0.046 (4)	0.008 (3)	−0.001 (3)	0.004 (3)
C16	0.060 (5)	0.050 (4)	0.050 (4)	0.000 (3)	0.010 (3)	0.005 (3)
C17	0.072 (5)	0.064 (4)	0.047 (4)	0.004 (4)	0.015 (4)	0.004 (3)
C18	0.063 (5)	0.057 (4)	0.060 (4)	−0.002 (4)	0.007 (4)	0.016 (4)
C19	0.072 (5)	0.043 (4)	0.065 (5)	0.002 (4)	0.009 (4)	0.007 (3)
C20	0.072 (5)	0.052 (4)	0.050 (4)	0.008 (4)	0.008 (4)	0.000 (3)
C21	0.056 (4)	0.052 (4)	0.032 (3)	−0.005 (3)	0.000 (3)	−0.004 (3)
C22	0.050 (4)	0.042 (3)	0.049 (4)	−0.002 (3)	0.009 (3)	−0.005 (3)
C23	0.050 (4)	0.043 (3)	0.040 (3)	−0.004 (3)	0.005 (3)	0.004 (3)
C24	0.057 (5)	0.063 (4)	0.045 (4)	−0.004 (4)	−0.005 (3)	0.004 (3)
C25	0.063 (5)	0.058 (4)	0.078 (5)	0.004 (4)	0.000 (4)	0.018 (4)
C26	0.079 (6)	0.039 (4)	0.090 (6)	0.005 (4)	0.001 (5)	0.007 (4)
C27	0.068 (5)	0.045 (4)	0.068 (5)	−0.002 (4)	0.005 (4)	−0.006 (3)
C28	0.116 (10)	0.164 (11)	0.078 (7)	−0.033 (9)	0.036 (7)	−0.039 (8)
C29	0.161 (13)	0.227 (15)	0.164 (12)	−0.055 (11)	−0.080 (10)	0.009 (11)
C30	0.131 (10)	0.120 (9)	0.125 (9)	−0.021 (8)	0.003 (8)	−0.019 (7)
C31	0.140 (6)	0.117 (6)	0.132 (6)	−0.012 (5)	0.040 (5)	−0.015 (5)
C32	0.143 (7)	0.123 (6)	0.115 (6)	0.015 (6)	0.022 (6)	−0.009 (5)
C33	0.200 (9)	0.201 (8)	0.205 (8)	−0.080 (7)	0.072 (7)	−0.001 (7)
C34	0.073 (5)	0.058 (5)	0.070 (5)	0.007 (4)	0.008 (4)	0.011 (4)
C35	0.180 (11)	0.069 (6)	0.115 (8)	−0.020 (6)	−0.012 (8)	−0.022 (5)
C36	0.104 (8)	0.084 (6)	0.120 (8)	0.017 (5)	0.020 (6)	0.017 (5)

### Geometric parameters (Å, °)

**Table:**

Nd1—O1^i^	2.341 (4)	C9—H9B	0.9700
Nd1—O2	2.344 (4)	C10—C11	1.512 (9)
Nd1—O3	2.448 (4)	C10—H10A	0.9700
Nd1—O3^i^	2.467 (4)	C10—H10B	0.9700
Nd1—N1^i^	2.616 (5)	C11—H11A	0.9700
Nd1—N2^i^	2.864 (4)	C11—H11B	0.9700
Nd1—N3	2.896 (5)	C12—C13	1.537 (9)
Nd1—N4	2.627 (5)	C12—H12A	0.9700
O1—C1	1.333 (7)	C12—H12B	0.9700
O1—Nd1^i^	2.341 (4)	C13—H13A	0.9700
O2—C15	1.328 (8)	C13—H13B	0.9700
O3—C22	1.364 (7)	C14—C16	1.459 (9)
O3—Nd1^i^	2.467 (4)	C14—H14	0.9300
O4—C28	1.227 (12)	C15—C20	1.441 (9)
O5—C31	1.272 (12)	C15—C16	1.442 (9)
O6—C34	1.217 (8)	C16—C17	1.446 (9)
N1—C7	1.301 (7)	C17—C18	1.388 (9)
N1—C8	1.494 (7)	C17—H17	0.9300
N1—Nd1^i^	2.616 (5)	C18—C19	1.414 (9)
N2—C10	1.525 (7)	C19—C20	1.401 (9)
N2—C9	1.527 (7)	C19—H19	0.9300
N2—C21	1.533 (7)	C20—H20	0.9300
N2—Nd1^i^	2.864 (4)	C21—C23	1.529 (8)
N3—C21	1.496 (8)	C21—H21	0.9800
N3—C12	1.512 (7)	C22—C27	1.415 (8)
N3—C11	1.533 (7)	C22—C23	1.436 (8)
N4—C14	1.304 (8)	C23—C24	1.415 (8)
N4—C13	1.492 (7)	C24—C25	1.403 (9)
N5—C28	1.353 (13)	C24—H24	0.9300
N5—C30	1.448 (12)	C25—C26	1.391 (9)
N5—C29	1.483 (12)	C26—C27	1.408 (9)
N6—C31	1.317 (12)	C26—H26	0.9300
N6—C32	1.456 (11)	C27—H27	0.9300
N6—C33	1.461 (13)	C28—H28	0.9300
N7—C34	1.343 (9)	C29—H29A	0.9600
N7—C36	1.457 (9)	C29—H29B	0.9600
N7—C35	1.476 (9)	C29—H29C	0.9600
Br1—C4	1.928 (7)	C30—H30A	0.9600
Br2—C18	1.936 (7)	C30—H30B	0.9600
Br3—C25	1.933 (7)	C30—H30C	0.9600
C1—C6	1.435 (8)	C31—H31	0.9300
C1—C2	1.453 (9)	C32—H32A	0.9600
C2—C3	1.414 (9)	C32—H32B	0.9600
C2—C7	1.471 (8)	C32—H32C	0.9600
C3—C4	1.409 (9)	C33—H33A	0.9600
C3—H3	0.9300	C33—H33B	0.9600
C4—C5	1.418 (9)	C33—H33C	0.9600
C5—C6	1.394 (10)	C34—H34	0.9300
C5—H5	0.9300	C35—H35A	0.9600
C6—H6	0.9300	C35—H35B	0.9600
C7—H7	0.9300	C35—H35C	0.9600
C8—C9	1.531 (8)	C36—H36A	0.9600
C8—H8A	0.9700	C36—H36B	0.9600
C8—H8B	0.9700	C36—H36C	0.9600
C9—H9A	0.9700		
			
O1^i^—Nd1—O2	132.23 (16)	C10—C11—H11A	110.3
O1^i^—Nd1—O3	141.78 (14)	N3—C11—H11A	110.3
O2—Nd1—O3	83.19 (14)	C10—C11—H11B	110.3
O1^i^—Nd1—O3^i^	82.36 (14)	N3—C11—H11B	110.3
O2—Nd1—O3^i^	143.18 (14)	H11A—C11—H11B	108.5
O3—Nd1—O3^i^	68.98 (14)	N3—C12—C13	113.0 (5)
O1^i^—Nd1—N1^i^	70.04 (15)	N3—C12—H12A	109.0
O2—Nd1—N1^i^	76.54 (15)	C13—C12—H12A	109.0
O3—Nd1—N1^i^	143.92 (14)	N3—C12—H12B	109.0
O3^i^—Nd1—N1^i^	112.44 (14)	C13—C12—H12B	109.0
O1^i^—Nd1—N4	75.43 (15)	H12A—C12—H12B	107.8
O2—Nd1—N4	70.15 (15)	N4—C13—C12	108.2 (5)
O3—Nd1—N4	112.86 (14)	N4—C13—H13A	110.0
O3^i^—Nd1—N4	142.50 (14)	C12—C13—H13A	110.0
N1^i^—Nd1—N4	87.97 (16)	N4—C13—H13B	110.0
O1^i^—Nd1—N2^i^	111.02 (14)	C12—C13—H13B	110.0
O2—Nd1—N2^i^	83.34 (14)	H13A—C13—H13B	108.4
O3—Nd1—N2^i^	83.07 (13)	N4—C14—C16	125.9 (6)
O3^i^—Nd1—N2^i^	70.15 (13)	N4—C14—H14	117.1
N1^i^—Nd1—N2^i^	65.33 (14)	C16—C14—H14	117.1
N4—Nd1—N2^i^	146.32 (15)	O2—C15—C20	120.1 (6)
O1^i^—Nd1—N3	82.21 (14)	O2—C15—C16	123.5 (6)
O2—Nd1—N3	110.68 (14)	C20—C15—C16	116.4 (6)
O3—Nd1—N3	69.76 (13)	C15—C16—C17	120.0 (6)
O3^i^—Nd1—N3	82.46 (13)	C15—C16—C14	123.1 (6)
N1^i^—Nd1—N3	145.60 (14)	C17—C16—C14	116.8 (6)
N4—Nd1—N3	65.05 (15)	C18—C17—C16	121.0 (6)
N2^i^—Nd1—N3	146.97 (14)	C18—C17—H17	119.5
O1^i^—Nd1—Nd1^i^	113.20 (11)	C16—C17—H17	119.5
O2—Nd1—Nd1^i^	114.57 (11)	C17—C18—C19	119.9 (6)
O3—Nd1—Nd1^i^	34.64 (9)	C17—C18—Br2	118.9 (5)
O3^i^—Nd1—Nd1^i^	34.34 (9)	C19—C18—Br2	121.2 (5)
N1^i^—Nd1—Nd1^i^	136.13 (11)	C20—C19—C18	120.0 (6)
N4—Nd1—Nd1^i^	135.87 (12)	C20—C19—H19	120.0
N2^i^—Nd1—Nd1^i^	73.76 (10)	C18—C19—H19	120.0
N3—Nd1—Nd1^i^	73.20 (10)	C19—C20—C15	122.5 (6)
C1—O1—Nd1^i^	136.9 (4)	C19—C20—H20	118.7
C15—O2—Nd1	135.7 (4)	C15—C20—H20	118.7
C22—O3—Nd1	120.3 (4)	N3—C21—C23	114.6 (5)
C22—O3—Nd1^i^	120.0 (4)	N3—C21—N2	105.6 (4)
Nd1—O3—Nd1^i^	111.02 (14)	C23—C21—N2	111.8 (5)
C7—N1—C8	117.1 (5)	N3—C21—H21	108.2
C7—N1—Nd1^i^	130.1 (4)	C23—C21—H21	108.2
C8—N1—Nd1^i^	112.8 (4)	N2—C21—H21	108.2
C10—N2—C9	111.6 (4)	O3—C22—C27	120.3 (5)
C10—N2—C21	102.1 (4)	O3—C22—C23	121.0 (5)
C9—N2—C21	108.6 (4)	C27—C22—C23	118.7 (6)
C10—N2—Nd1^i^	107.0 (3)	C24—C23—C22	118.9 (6)
C9—N2—Nd1^i^	108.6 (3)	C24—C23—C21	119.8 (5)
C21—N2—Nd1^i^	118.9 (3)	C22—C23—C21	121.3 (5)
C21—N3—C12	108.8 (5)	C25—C24—C23	120.9 (6)
C21—N3—C11	102.9 (5)	C25—C24—H24	119.6
C12—N3—C11	112.4 (5)	C23—C24—H24	119.6
C21—N3—Nd1	118.6 (3)	C26—C25—C24	120.6 (6)
C12—N3—Nd1	108.3 (4)	C26—C25—Br3	120.2 (5)
C11—N3—Nd1	105.8 (3)	C24—C25—Br3	119.2 (5)
C14—N4—C13	117.8 (5)	C25—C26—C27	119.6 (6)
C14—N4—Nd1	129.4 (4)	C25—C26—H26	120.2
C13—N4—Nd1	112.8 (4)	C27—C26—H26	120.2
C28—N5—C30	119.7 (9)	C26—C27—C22	121.2 (6)
C28—N5—C29	120.8 (11)	C26—C27—H27	119.4
C30—N5—C29	119.4 (10)	C22—C27—H27	119.4
C31—N6—C32	119.4 (10)	O4—C28—N5	126.5 (11)
C31—N6—C33	116.9 (10)	O4—C28—H28	116.7
C32—N6—C33	122.9 (10)	N5—C28—H28	116.7
C34—N7—C36	121.1 (7)	N5—C29—H29A	109.5
C34—N7—C35	121.2 (7)	N5—C29—H29B	109.5
C36—N7—C35	117.6 (7)	H29A—C29—H29B	109.5
O1—C1—C6	120.1 (6)	N5—C29—H29C	109.5
O1—C1—C2	122.9 (5)	H29A—C29—H29C	109.5
C6—C1—C2	117.0 (6)	H29B—C29—H29C	109.5
C3—C2—C1	119.5 (6)	N5—C30—H30A	109.5
C3—C2—C7	118.1 (6)	N5—C30—H30B	109.5
C1—C2—C7	122.2 (6)	H30A—C30—H30B	109.5
C4—C3—C2	122.2 (7)	N5—C30—H30C	109.5
C4—C3—H3	118.9	H30A—C30—H30C	109.5
C2—C3—H3	118.9	H30B—C30—H30C	109.5
C3—C4—C5	118.4 (7)	O5—C31—N6	125.3 (12)
C3—C4—Br1	121.0 (6)	O5—C31—H31	117.3
C5—C4—Br1	120.6 (5)	N6—C31—H31	117.3
C6—C5—C4	120.8 (6)	N6—C32—H32A	109.5
C6—C5—H5	119.6	N6—C32—H32B	109.5
C4—C5—H5	119.6	H32A—C32—H32B	109.5
C5—C6—C1	122.0 (7)	N6—C32—H32C	109.5
C5—C6—H6	119.0	H32A—C32—H32C	109.5
C1—C6—H6	119.0	H32B—C32—H32C	109.5
N1—C7—C2	126.6 (6)	N6—C33—H33A	109.5
N1—C7—H7	116.7	N6—C33—H33B	109.5
C2—C7—H7	116.7	H33A—C33—H33B	109.5
N1—C8—C9	107.7 (5)	N6—C33—H33C	109.5
N1—C8—H8A	110.2	H33A—C33—H33C	109.5
C9—C8—H8A	110.2	H33B—C33—H33C	109.5
N1—C8—H8B	110.2	O6—C34—N7	125.4 (7)
C9—C8—H8B	110.2	O6—C34—H34	117.3
H8A—C8—H8B	108.5	N7—C34—H34	117.3
N2—C9—C8	112.8 (5)	N7—C35—H35A	109.5
N2—C9—H9A	109.0	N7—C35—H35B	109.5
C8—C9—H9A	109.0	H35A—C35—H35B	109.5
N2—C9—H9B	109.0	N7—C35—H35C	109.5
C8—C9—H9B	109.0	H35A—C35—H35C	109.5
H9A—C9—H9B	107.8	H35B—C35—H35C	109.5
C11—C10—N2	107.0 (5)	N7—C36—H36A	109.5
C11—C10—H10A	110.3	N7—C36—H36B	109.5
N2—C10—H10A	110.3	H36A—C36—H36B	109.5
C11—C10—H10B	110.3	N7—C36—H36C	109.5
N2—C10—H10B	110.3	H36A—C36—H36C	109.5
H10A—C10—H10B	108.6	H36B—C36—H36C	109.5
C10—C11—N3	107.3 (5)		
			
O1^i^—Nd1—O2—C15	7.2 (6)	C3—C2—C7—N1	−170.8 (6)
O3—Nd1—O2—C15	−156.6 (5)	C1—C2—C7—N1	14.0 (10)
O3^i^—Nd1—O2—C15	163.1 (5)	C7—N1—C8—C9	117.0 (6)
N1^i^—Nd1—O2—C15	53.5 (5)	Nd1^i^—N1—C8—C9	−60.6 (5)
N4—Nd1—O2—C15	−39.2 (5)	C10—N2—C9—C8	85.1 (6)
N2^i^—Nd1—O2—C15	119.7 (5)	C21—N2—C9—C8	−163.2 (4)
N3—Nd1—O2—C15	−91.2 (5)	Nd1^i^—N2—C9—C8	−32.6 (5)
Nd1^i^—Nd1—O2—C15	−171.6 (5)	N1—C8—C9—N2	62.4 (6)
O1^i^—Nd1—O3—C22	−103.7 (4)	C9—N2—C10—C11	139.9 (5)
O2—Nd1—O3—C22	56.8 (4)	C21—N2—C10—C11	24.1 (6)
O3^i^—Nd1—O3—C22	−147.8 (5)	Nd1^i^—N2—C10—C11	−101.5 (5)
N1^i^—Nd1—O3—C22	112.6 (4)	N2—C10—C11—N3	−2.2 (6)
N4—Nd1—O3—C22	−8.3 (4)	C21—N3—C11—C10	−21.2 (6)
N2^i^—Nd1—O3—C22	140.9 (4)	C12—N3—C11—C10	−138.1 (5)
N3—Nd1—O3—C22	−58.2 (4)	Nd1—N3—C11—C10	103.9 (5)
Nd1^i^—Nd1—O3—C22	−147.8 (5)	C21—N3—C12—C13	163.0 (5)
O1^i^—Nd1—O3—Nd1^i^	44.1 (3)	C11—N3—C12—C13	−83.7 (6)
O2—Nd1—O3—Nd1^i^	−155.44 (16)	Nd1—N3—C12—C13	32.8 (6)
O3^i^—Nd1—O3—Nd1^i^	0.0	C14—N4—C13—C12	−118.4 (6)
N1^i^—Nd1—O3—Nd1^i^	−99.6 (2)	Nd1—N4—C13—C12	60.2 (6)
N4—Nd1—O3—Nd1^i^	139.49 (15)	N3—C12—C13—N4	−62.7 (7)
N2^i^—Nd1—O3—Nd1^i^	−71.36 (15)	C13—N4—C14—C16	173.0 (6)
N3—Nd1—O3—Nd1^i^	89.54 (16)	Nd1—N4—C14—C16	−5.3 (10)
O1^i^—Nd1—N3—C21	156.1 (4)	Nd1—O2—C15—C20	−145.5 (5)
O2—Nd1—N3—C21	−71.6 (4)	Nd1—O2—C15—C16	35.8 (9)
O3—Nd1—N3—C21	2.5 (4)	O2—C15—C16—C17	−179.6 (6)
O3^i^—Nd1—N3—C21	72.8 (4)	C20—C15—C16—C17	1.7 (9)
N1^i^—Nd1—N3—C21	−168.0 (3)	O2—C15—C16—C14	3.9 (10)
N4—Nd1—N3—C21	−126.4 (4)	C20—C15—C16—C14	−174.8 (6)
N2^i^—Nd1—N3—C21	39.1 (5)	N4—C14—C16—C15	−16.1 (11)
Nd1^i^—Nd1—N3—C21	38.9 (3)	N4—C14—C16—C17	167.3 (7)
O1^i^—Nd1—N3—C12	−79.4 (4)	C15—C16—C17—C18	1.5 (10)
O2—Nd1—N3—C12	52.9 (4)	C14—C16—C17—C18	178.2 (6)
O3—Nd1—N3—C12	127.0 (4)	C16—C17—C18—C19	−3.6 (10)
O3^i^—Nd1—N3—C12	−162.7 (4)	C16—C17—C18—Br2	176.0 (5)
N1^i^—Nd1—N3—C12	−43.5 (5)	C17—C18—C19—C20	2.4 (10)
N4—Nd1—N3—C12	−1.9 (4)	Br2—C18—C19—C20	−177.2 (5)
N2^i^—Nd1—N3—C12	163.6 (3)	C18—C19—C20—C15	1.0 (10)
Nd1^i^—Nd1—N3—C12	163.4 (4)	O2—C15—C20—C19	178.3 (6)
O1^i^—Nd1—N3—C11	41.3 (4)	C16—C15—C20—C19	−2.9 (10)
O2—Nd1—N3—C11	173.6 (4)	C12—N3—C21—C23	−80.3 (6)
O3—Nd1—N3—C11	−112.3 (4)	C11—N3—C21—C23	160.4 (5)
O3^i^—Nd1—N3—C11	−42.0 (4)	Nd1—N3—C21—C23	44.0 (5)
N1^i^—Nd1—N3—C11	77.3 (4)	C12—N3—C21—N2	156.3 (4)
N4—Nd1—N3—C11	118.8 (4)	C11—N3—C21—N2	36.9 (5)
N2^i^—Nd1—N3—C11	−75.7 (4)	Nd1—N3—C21—N2	−79.4 (4)
Nd1^i^—Nd1—N3—C11	−75.9 (3)	C10—N2—C21—N3	−38.3 (5)
O1^i^—Nd1—N4—C14	−123.5 (6)	C9—N2—C21—N3	−156.2 (4)
O2—Nd1—N4—C14	22.8 (6)	Nd1^i^—N2—C21—N3	79.1 (4)
O3—Nd1—N4—C14	96.0 (6)	C10—N2—C21—C23	−163.5 (5)
O3^i^—Nd1—N4—C14	−179.2 (5)	C9—N2—C21—C23	78.5 (5)
N1^i^—Nd1—N4—C14	−53.6 (6)	Nd1^i^—N2—C21—C23	−46.2 (6)
N2^i^—Nd1—N4—C14	−17.4 (7)	Nd1—O3—C22—C27	−105.6 (6)
N3—Nd1—N4—C14	148.4 (6)	Nd1^i^—O3—C22—C27	109.5 (6)
Nd1^i^—Nd1—N4—C14	128.0 (5)	Nd1—O3—C22—C23	73.8 (7)
O1^i^—Nd1—N4—C13	58.1 (4)	Nd1^i^—O3—C22—C23	−71.1 (6)
O2—Nd1—N4—C13	−155.6 (5)	O3—C22—C23—C24	178.3 (6)
O3—Nd1—N4—C13	−82.4 (4)	C27—C22—C23—C24	−2.3 (9)
O3^i^—Nd1—N4—C13	2.5 (5)	O3—C22—C23—C21	−2.6 (9)
N1^i^—Nd1—N4—C13	128.0 (4)	C27—C22—C23—C21	176.8 (6)
N2^i^—Nd1—N4—C13	164.3 (4)	N3—C21—C23—C24	121.4 (6)
N3—Nd1—N4—C13	−30.0 (4)	N2—C21—C23—C24	−118.5 (6)
Nd1^i^—Nd1—N4—C13	−50.3 (5)	N3—C21—C23—C22	−57.7 (7)
Nd1^i^—O1—C1—C6	144.0 (5)	N2—C21—C23—C22	62.5 (7)
Nd1^i^—O1—C1—C2	−37.2 (9)	C22—C23—C24—C25	0.9 (10)
O1—C1—C2—C3	−176.0 (6)	C21—C23—C24—C25	−178.2 (6)
C6—C1—C2—C3	2.9 (9)	C23—C24—C25—C26	2.1 (11)
O1—C1—C2—C7	−0.9 (9)	C23—C24—C25—Br3	−179.1 (5)
C6—C1—C2—C7	178.0 (6)	C24—C25—C26—C27	−3.6 (12)
C1—C2—C3—C4	−3.6 (10)	Br3—C25—C26—C27	177.6 (6)
C7—C2—C3—C4	−178.9 (6)	C25—C26—C27—C22	2.2 (12)
C2—C3—C4—C5	1.8 (10)	O3—C22—C27—C26	−179.8 (6)
C2—C3—C4—Br1	−177.1 (5)	C23—C22—C27—C26	0.7 (10)
C3—C4—C5—C6	0.5 (11)	C30—N5—C28—O4	0.9 (17)
Br1—C4—C5—C6	179.5 (6)	C29—N5—C28—O4	178.1 (12)
C4—C5—C6—C1	−1.1 (11)	C32—N6—C31—O5	−173.4 (12)
O1—C1—C6—C5	178.3 (6)	C33—N6—C31—O5	17.0 (19)
C2—C1—C6—C5	−0.6 (10)	C36—N7—C34—O6	2.7 (12)
C8—N1—C7—C2	−172.8 (6)	C35—N7—C34—O6	−176.7 (8)
Nd1^i^—N1—C7—C2	4.4 (9)		

Symmetry codes: (i) −*x*+2, −*y*+1, −*z*+2.

### Hydrogen-bond geometry (Å, °)

**Table:**

*D*—H···*A*	*D*—H	H···*A*	*D*···*A*	*D*—H···*A*
C21—H21···O4	0.98	2.58	3.514 (10)	159
